# Clinical Results 5 Years after Great Saphenous Vein Stripping

**DOI:** 10.3400/avd.oa.21-00020

**Published:** 2021-06-25

**Authors:** Hitoshi Kusagawa, Yasuhisa Ozu, Kentaro Inoue, Takuya Komada, Yoshihiko Katayama

**Affiliations:** 1 Matsusaka Ohta Clinic, Matsusaka, Mie, Japan; 2 Department of Thoracic and Cardiovascular Surgery, Matsusaka Chuo General Hospital, Matsusaka, Mie, Japan

**Keywords:** GSV stripping, recurrent varices after surgery (REVAS), mid-term results, high ligation, neovascularization

## Abstract

**Background:** As a standard treatment for the varicose vein of the great saphenous vein (GSV) type, endovenous ablation (EVA) is the main approach. However, as a background to this, in Europe and the United States, neovascularization (Neo) following high ligation (HL) of the saphenofemoral junction (SFJ) at the time of GSV stripping has been emphasized as one of the reasons for the high rate of recurrence. However, in Japan, almost no similar mid- or long-term results of GSV stripping have been reported.

**Patients and Methods:** From September 2011 to March 2014 when EVA was not my surgical option, 413 consecutive legs of patients underwent GSV stripping by myself using the same procedure. The patients were contacted by phone 5 years later, and recurrent varices after surgery (REVAS) and reoperation (REDO) were investigated. A total of 270 legs of the 391 living cases (69%) underwent venous ultrasonography (VUS). HL of the SFJ was performed via central flash ligation with towing and pulling out of the peripheral side branches containing the accessory saphenous veins. In principle, GSV stripping was performed using the invagination method in the range of the entire reflux region from the HL cut section to the confluent section of the side branch causing branch varicose veins. The range of stripping was to the upper thigh in 3 legs, to the middle thigh in 3 legs, to the lower thigh in 7 legs, to the knee in 46 legs, to the upper calve in 83 legs, to the middle calve in 52 legs, and over the full length in 76 legs. Stab avulsion was performed as much as possible for the side-branch varices. On VUS, the SFJ’s stump of GSV, the presence of side-branch remnants and their reflux, the presence or absence of Neo, and the recurrent lesions in other sites were evaluated. REVAS were classified as follows: Level 1, symptomatic recurrent lesion for which surgery is indicated; Level 2, asymptomatic recurrent lesion possibly requiring future surgery; and Level 3, asymptomatic recurrent lesion that is unlikely to require future surgery.

**Results:** Of the 391 legs of patients who could be contacted, REDO was performed in 23 (6%), including 15 limbs, immediately after this investigation, and symptomatic REVAS were observed in 29 (7%). In 270 legs examined by VUS, REVAS were diagnosed as follows: 29 legs with Level 1 lesion, 40 legs with Level 2 lesion, and 27 legs with Level 3 lesion. Level 1 REVAS that occurred at the SFJ were observed only in 3 legs (1.1%), Level 1 REVAS due to incompetent perforating veins (IPVs) were observed in 14 legs (5%), and Level 1 solitary tributary varices were observed in 9 legs (3%).

**Conclusion:** In this study, REVAS at the SFJ were significantly less than those in the past reports. It has been shown that REVAS due to IPVs or solitary tributary varices were more important than those at the SFJ. (This is a translation of Jpn J Phlebol 2019; 30(3): 259–265.)

## Introduction

As the standard surgery for varicose veins of the great saphenous vein (GSV) type, endovenous ablation (EVA) is employed instead of the traditional stripping (STR). Moreover, in the Western guideline^1)^ for the treatment, the recommendation level of EVA is higher than that of STR owing to its good initial results. In Japan, after the acceptance of EVA as a medical service covered by the national health insurance in 2011, such tendency towards the endovenous treatments has become remarkable. As one of the reasons, Western countries have emphasized high recurrence rate due to neovascularization (Neo) on the saphenofemoral junction (SFJ) following high ligation (HL) in the stripping technique, and the recurrence rate increases over time.^2–4)^ However, recurrence caused by Neo is rare from my experience.^5)^ Thus, the tone of article that GSV-STR is not good surgery because of high rate of recurrence at SFJ in Western countries made me feel uncomfortable. As the mid- and long-term results of GSV-STR were almost not observed in Japan, the clinical results 5 years following GSV stripping were elucidated in this paper.

## Patient Population and Methods

**Figure 1:**
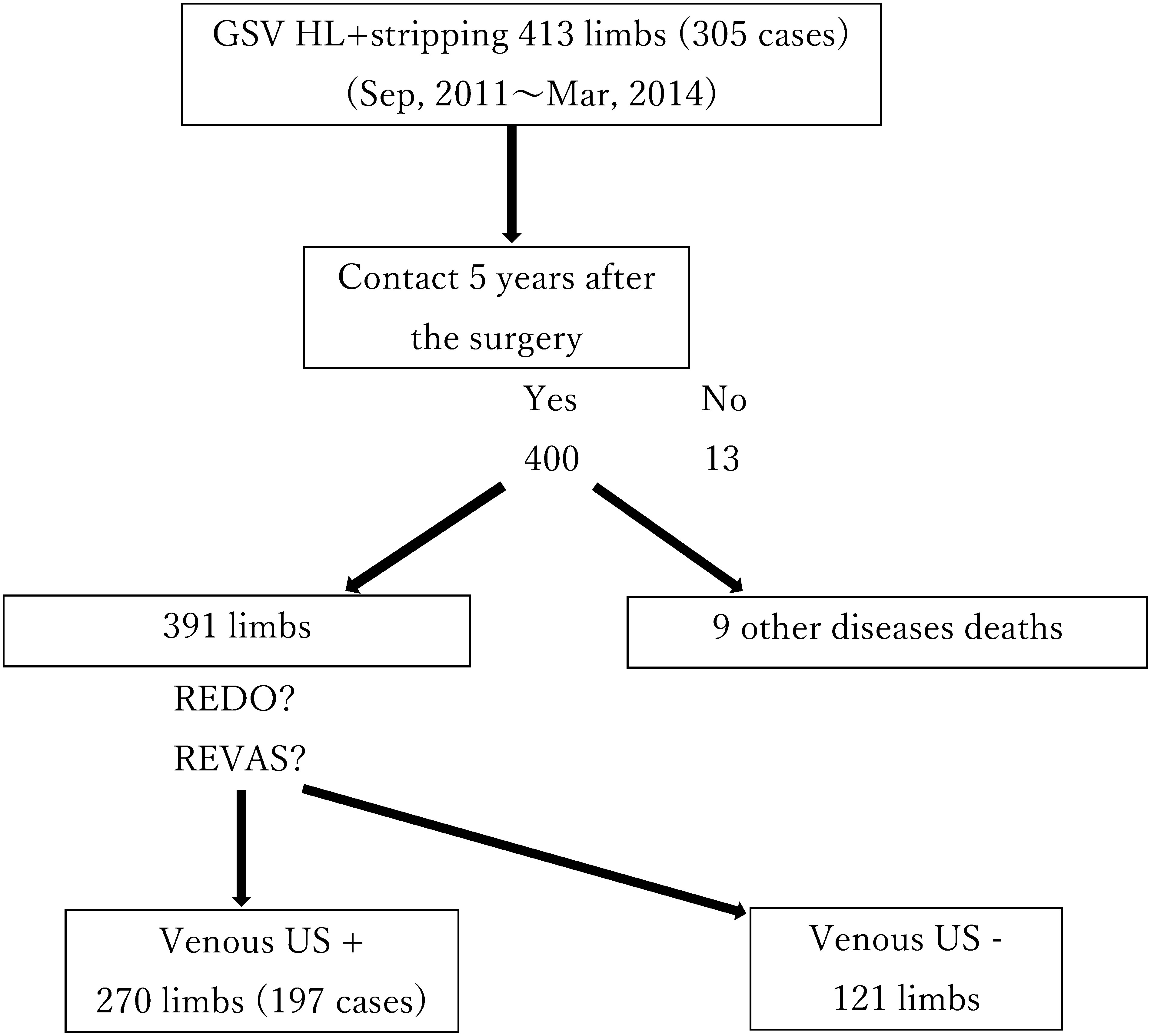
Fig. 1 Chart of the study.

The chart of this study is presented in **Fig. 1**. From September 2011 to March 2014, when EVA was still not my alternative strategy for the treatment of varicose veins, consecutive 413 legs of 305 cases underwent GSV-STR with HL at the SFJ by myself. I attempted to contact these patients by telephone 5 years after the surgery, and I was able to obtain information of 400 legs. Six patients with 9 legs already died of other diseases, and in the remaining 391 legs, the existence of reoperation (REDO) and recurrent varices after surgery (REVAS) was inquired. With regard to the definition of Perrin et al. of REVAS,^6)^ the patients were asked about their awareness of REVAS, the recurrence of preoperative symptoms, and the existence of venous statis dermatitis. Of the 391 legs contacted, 270 legs of 197 cases (69%) came to the clinic to investigate with venous ultrasongraphy (VUS). In the remaining 121 legs of patients without VUS, no REDO and 3 REVAS occurred, which were regarded as asymptomatic branch varicose veins by listening on the phone. The characteristics of the patients who underwent VUS are presented in **Table 1**. There were 65 males and 132 females, and their average age during surgery was 65.0±9.6 years. Their clinical stage on the Clinical, Etiologic Anatomic Pathophysiologic (CEAP) classification was distributed into 118 C2s, 19 C3s, 85 C4as, 31 C4bs, 6 C5s, and 11 C6s. As simultaneous surgeries, 44 direct incompetent perforating vein (IPV) severings, 30 subfascial endoscopic perforator surgeries (SEPSs), and 21 small saphenous veins (SSV) STRs were performed.

REVAS: recurrent varices after surgery

**Table 1:** Table 1 Characteristics of the patients who underwent  venous ultrasonography

	Total 270 limbs, 197 cases
Age	65.0±9.6
Male/Female	65/132
CEAP	
C2	118
C3	19
C4a	85
C4b	31
C5	6
C6	11
Follow-up period (months)	60.1±2.5 (46～75)
Simultaneous surgery	
Direct IPV severing	44
SEPS	30
SSV stripping	21

IPV: incompetent perforating vein; SEPS: subfascial endoscopic perforator surgery; SSV: small saphenous vein

The same surgeon (HK) performed GSV-STR using the same procedure, i.e., HL at the SFJ was done as follows: GSV was identified and divided and severed through 1–1.5 cm of skin incision on inguinal crease, observing with headlight and magnifying loupe. Subsequently, the proximal stump was doubly ligated on the junction after all tributaries at the SFJ were ligated, and peripheral tributaries were pulled out after being divided by local anesthesia injection and metal ware, not to cause recurrence by Neo as much as possible, according to the method described by Kokubo et al.^7)^ Stripping was performed between the peripheral cut edge of HL and the GSV line connected by the lowest tributary, which caused the varicose veins to occur, basically using the invagination method. The range of stripping was to the upper thigh in 3 legs, to the middle thigh in 3 legs, to the lower thigh in 7 legs, to the knee in 46 legs, to the upper calf in 83 legs, to the middle thigh in 52 legs, and over the full length in 76 legs. Tributary varicosities were removed using the stab avulsion technique as much as possible. It was performed in 408 out of 413 legs.

In the VUS, the residual GSV stump at the SFJ, its connecting tributaries, Neo, other sites were observed, and refluxing lesions were examinated via Doppler spectroscopy. With regard to Neo, the diagnostic condition was decided to be 0 mm of the stump length or not continuous with the SFJ. REVAS were defined as follows: Level 1, the REVAS state with reflux that needed REDO, i.e., symptomatic state, or varicose veins ≧3 mm in diameter or symptomatic; Level 2, recurrent lesions likely to be indicated for REDO in the future, i.e., asymptomatic clear reflux lesions, or varicose veins <3 mm in diameter and that are asymptomatic; Level 3, recurrent lesions unlikely to be indicated for REDO in the future, i.e., extremely mild and small-diameter reflux, or varicose veins <2 mm in diameter and that are asymptomatic, or Neo-localized below the superficial fascia, whose reflux was not clear. In the VUS performed on the legs, saphenous neuropathy was also evaluated.

## Results

By listening on the phone to the 391 legs, it was found that REDO were performed in 23 legs (6%), including 15 legs after this survey, REVAS were in 29 legs (7%).

All refluxing veins that caused REDO and REVAS per the Level in 270 legs, which had VUS (69%) are presented in **Table 2**. Although 29 (11%) Level 1 lesions, 40 (15%) Level 2 lesions, and 27 (10%) Level 3 lesions were observed, these numbers shown were the total numbers. There were many cases who had multiple lesions on the same leg. Although Neo around the SFJ was observed in 22 legs (8%), 15 of these legs were of Level 3 and were unlikely to be indicated for REDO in the future. Moreover, they were localized below the superficial fascia, whose reflux was not clear. Only 2 of these legs (both sides of the same case, REDO not performed yet) were of Level 1, which was likely to be indicated for REDO, and 5 were of Level 2, asymptomatic slight reflux lesions, which extended to subcutaneous tissue likely to be worsen and indicated for REDO in the future. Another cause of SFJ recurrence, reflux lesion of tributaries from the SFJ stump was observed only in one leg which was of Level 1 (REDO already done). Thus, only 3 legs of Level 1 (1.1%) and 9 legs (3.3%) of Level 2 REVAS related to the SFJ were identified.

**Table 2:** Table 2 Results of venous ultrasonography of 268 limbs-part 1

Origin	→	IPV	SSV	Branch	Calf GSV	SFJ-branch	SPJ-branch	Neo
REDO	23 legs	10	6	11	2	1	1	0
REVAS								
Level 1	29 legs	14	7	9	4	1	1	2
Level 2	40 legs	13	4	11	6	1	0	5
Level 3	27 legs	6	0	6	5	1	0	15

IPV: incompetent perforating vein; SSV: small saphenous vein; GSV: great saphenous vein; SFJ: sapheno-femoral junction; SPJ: sapheno-popliteal junction; Neo: neovascularization; REVAS: recurrent varices after surgery

The most common cause of Level 1 and 2 REVAS was IPV, followed by branch varices. REVAS related to SSV, calf GSV, and SFJ were less.

The findings of SFJ from the VUS results are presented in **Table 3**. The average stump length was 1.2±2.5 mm (0–11.3 mm, 0 mm in 198 legs, 73%), and tributaries connecting to the residual stump were observed in 55 legs (20%), including the accessory saphenous vein in 16 legs (6%). Refluxing tributaries were observed only in two legs.

**Table 3:** Table 3 Results of venous ultrasonography of 270 limbs- part 2

SFJ-finding	
Stump length (mm)	1.2±2.5 (0～11.3)
Residual branch	55 (20%)
Residual accessory SV	16 (6%)
Refluxing residual accessory SV	2 (0.7%)
Neovascularization	22 (8%)
Level 1	2
Level 2	5
Level 3	15

SV: saphenous vein

Finally, the evaluation of saphenous neuropathy following surgery in 270 legs that underwent VUS is presented in **Table 4**. Saphenous neuropathy was observed in 29 legs (11%); however, no patient complained that it interfered with their daily lives. As previously pointed out, neuropathy was often observed during STRs performed on the peripheral side of the leg; 17% of the STRs were performed on the middle of the lower leg, whereas 22% were performed over the full length of the leg. However, the absolute ratio tended to be lower than those of the previous reports; however, it may be a relatively long 5 years.

**Table 4:** Table 4 Results of venous ultrasonography of 268 limbs- part 3

Range of GSV stripping	Saphenous nerve injury/total legs
To upper thigh	0/3 (0%)
To middle thigh	0/3 (0%)
To lower thigh	0/7 (0%)
To the knee	2/46 (4%)
To upper calf	1/83 (1%)
To middle calf	9/52 (17%)
Full	17/76 (22%)
Total	29/270 (11%)
Adverse effects on daily living	0/270 (0%)

## Discussion

There is no doubt about the good early results of EVA for the treatment of GSV. Moreover, the high occlusion rate and few complications of EVA indicate its high reliability for therapeutic devices, and the level of the guideline recommendation in the United States and Europe is higher in EVA than that in STR.^1)^ EVA is also much easier, simpler, and quicker for surgeons to perform than STR. However, there are many unclear points with regard to the mid- to long-term results.

One of the reasons why EVA has been widely used instead of GSV-STR is that, with GSV-STR, there is a high and increasing recurrence rate due to Neo around the deep vein junction which occurs after HL. This phenomenon, as described in numerous Western papers, is inevitable with this surgical procedure. With regard to the incidence of REVAS, Earnshaw’s group demonstrated that 25% 2 years after HL+stripping (HL+S),^2)^ 35% 5 years,^3)^ 62% 11 years,^4)^ of which REDO was 6% 5 years,^3)^ 21% in 11 years,^4)^ whereas Neo diagnosis rate was 45% 2 years,^2)^ 23% 5 years,^3)^ and 65% 11 years,^4)^ which was a very high value. Fischer et al.^8)^ described 60% inguinal REVAS and 36% REDO 34 years following surgery. In the report by Hartmann et al.,^9)^ the incidence rate of REVAS of the groin 14 years after surgery was 40%. In these Western reports^2–4,8,9)^ the SFJ stump tributary regurgitation due to technical failure and so-called Neo were treated as a jumble. Most of these reports were published in the 1990s when no high-quality VUS existed during the initial surgeries. Therefore, it seems that diagnostic level was also low. For this reason, the theory of asserting the importance of Neo’s REVAS on the premise of 100% success was developed without evaluating the technical success following surgery, and accurate evaluation and diagnosis of the progress seemed to be difficult. In addition, in the papers in which the incidence of REVAS was followed by surgical treatment of GSV and SSV, 25% to 50% of REVAS occurred after 3 to 5 years, including those with no symptoms,^6,10–12)^ and 18% after 5 years with symptoms.^10)^ Moreover, 29% of REVAS due to Neo occurred after 5 years,^10)^ 24% after 3 years,^11)^ and 5%–25% after 5 years.^12)^ However, it was not mentioned in the reports how many of it required REDO. On the other hand, the rate of REDO for REVAS in our past cases of varicose vein surgery was 7%, especially Neo was 0.3%, which was very small,^5)^ and was inconsistent with the previous reports in the United States and Europe. In addition, the mid- to long-term results of GSV-STR in Japan were hardly demonstrated. Therefore, this time, we investigated the clinical results of our own GSV-STR after 5 years and examined REDO, REVAS, SFJ stump mainly on VUS. REVAS with symptom were found in 7%, Neo in 0.5%. REVAS with possible future symptom due to deterioration were found in 10%, of which only 1.3% is due to Neo. These results were quite different from that of Europe and the United States.

Neo was originally proposed by Glass^13)^ as a finding of venography in 1987, and a unified view on its definition and pathological condition has not yet been obtained. There are two hypotheses about its actual situation. One is the idea that, literally, the venules of granulation tissue formed in the dead space after surgery are connected to the venules left in the surroundings, which results in the prolongation of REVAS as new blood vessels.^14,15)^ The other is that it is not a new blood vessel, but the originally existing venous channel is connected via the SFJ lateral branch left behind following surgery.^14,16)^ In any case, it seems that the seeds of REVAS due to Neo do not appear suddenly after many years, but shapes of that are completed relatively early after surgery and can be observed via VUS, and only a small part of them deteriorate and become apparent in the future. In fact, in VUS 6 months after surgery in another HL+S case group, Neo was detected in 8% of all legs (unpublished). This rate was almost the same as that of this study 5 years after surgery. Although Neo was recognized in VUS this time as described above, 68% of them the Neo lesions were localized under the superficial fascia and had no clear reflux. Recently, in Europe, data suggesting that side-branch regurgitation from the SFJ stump left following HT+S or EVA resulted in a significantly higher rate of symptomatic REVAS than Neo.^17–19)^ There are reports in immunohistological studies^20)^ denying Neo as a neovascularization. The other report demonstrated that it is a mere innocent bystander unrelated to REVAS.^21)^

Compared to our results, the reasons why the incidences of the REVAS and Neo were higher in the reports in Europe and the United States are thought to be differences of diagnosis level in VUS, differences in the procedure of HL, and differences by race and physique.

First, with regard to the diagnosis of VUS, including the diagnosis of REVAS, a large difference in diagnostic level can be observed among the practitioners. Especially for Neo, the actual condition is not clear as described above, and the diagnosis itself is ambiguous. Therefore, for the sake of simplicity, Neo was uniformly defined to be small veins near 0 mm length of the SFJ stump or small veins near the SFJ stump with no backflow tributaries. Thus, in all cases, the GSV stump length of the SFJ following HL was measured via VUS and observed and evaluated in detail. This was done to completely eliminate the reflux from the tributaries of the SFJ stump, that is, the technical failure. With regard to HL, as in Kokubo’s idea,^7)^ I consider that if the peripheral tributaries around the SFJ remain, REVAS may occur by itself or in relation to Neo. For the above reasons, the peripheral tributaries were pulled out as much as possible according to Kokubo’s method^7)^ and this maneuver is completely different from many reports in Europe and the United States. Even in Europe and the United States, Stonebridge et al.^22)^ and Bergan et al.^23)^ similarly mentioned the possibility of suppressing REVAS near the SFJ via “extended tributary resection”; however, it was not widely used. It has also been suggested that one of the causes of the high incidence of REVAS is that it is difficult to accurately perform HL in Westerners due to the higher rate of obesity compared with the Japanese, but this case group also included a person with a body weight of 120 to 130 kg, and he was able to undergo HL at 4 to 5 cm depth of SFJ without leaving a stump via a 2 cm of skin incision. Even in such cases, the depth at which GSV was identified and dissociation was performed is at most about 2 cm. It is not difficult for a skilled surgeon to process the tributaries by separating the surroundings from the proximal GSV toward the SFJ, although it is time-consuming. Based on experience, it is considered that the degree of inflammatory adhesion around the vein near the SFJ rather than the depth makes the separation of tissues difficult. Moreover, the fragile vein wall is more likely to be affected by the procedure, thus making HL difficult to perform. During tumescent local anesthesia performed during EVA, injection of the anesthetic solution into the saphenous compartment of the thigh easily separates the liquid around the GSV; however, when injected close to the SFJ in some cases, the liquid is not easily separated. In such cases, accurate HL will be difficult to perform, and EVA will be significantly superior than HT+S in such a case.

If the HL+S technique itself is evaluated, the results of the cases with minimally successful surgery need to be considered. But in Western papers, no mention was made of the evaluation of whether the surgery was technically successful and no definition of technical success was given. If the technical success is defined that the stump length of SFJ is 0 mm, the surgical success rate in this study was 73% in 198 of 270 legs. There were naturally no REVAS due to reflux from the SFJ stump to tributaries in these technically succeeded legs. Among 198 technically succeed legs, symptomatic REVAS occurred in 17 legs (9%), but no Neo was observed in the causative vein. As in all cases, there were nine cases of IPV and seven cases of side-branch varicose veins alone, which were by far the most.

It has also been suggested that the standard treatment for varicose veins is HT+S in some countries in Europe where patients are burdened with EVA.^24)^ In a new European meta-analysis of GSV,^25)^ the 5-year REVAS rate was 12% following HL+S and 22% following EVA, which was significantly better for HL+S. The main cause of REVAS following EVA is tributary reflux from the SFJ stump, which is also observed in technically failed HL.^26,27)^ With reference to the results of the GSV HL+S after 5 years, we will examine the results of our own EVA and follow up the 10-year results of this STR group of patients, focusing on the cases that were judged to be of Level 2 Neo this time. Moreover, I will use the results to decide on the treatment policy at our institution. I hopes that this study will be useful for varicose vein surgeons and co-medical staff of the next generation of endovenous treatment development who will be required to diagnose REVAS and to select treatment strategy against them.

## Conclusion

The clinical results 5 years after GSV-STR of 413 limbs in 305 cases were summarized. REVAS with clinical symptoms were recognized in 7%, and REDO was indicated in 6%. However, symptomatic REVAS derived from SFJ were as small as 1.1%, and most of REVAS had IPV or superficial branch varicose veins. The results were quite different from those demonstrated in the Western countries.

## Additional Note

The content of this manuscript was presented on Symposium at the 38th Annual Meeting of the Japanese Society of Phlebology in June 2018 in Yokosuka, Japan.

## Notes

This is a translation of Jpn J Phlebol 2019; 30(3): 259–265.

## References

[1] Gloviczki P, Comerota AJ, Dalsing MC, et al. The care of patients with varicose veins and associated chronic venous diseases: clinical practice guidelines of the Society for Vascular Surgery and American Venous Forum. J Vasc Surg 2011; 53 **Suppl**: 2S-48S. 21536172 10.1016/j.jvs.2011.01.079

[2] Jones L, Braithwaite BD, Selwyn D, et al. Neovascularization is the principal cause of varicose vein recurrence: results of randomized trial of stripping the long saphenous vein. Eur J Vasc Endovasc Surg 1996; 12: 442-5. 8980434 10.1016/s1078-5884(96)80011-6

[3] Dwerryhouse S, Davies B, Harradine K, et al. Stripping the long saphenous vein reduces the rate of reoperation for recurrent varicose vein: five year results of a randomized trial. J Vasc Surg 1999; 29: 589-92. 10194484 10.1016/s0741-5214(99)70302-2

[4] Winterborn RJ, Foy C, Earnshaw JJ. Cause of varicose vein recurrence: late results of a randomized controlled trial of stripping the long saphenous vein. J Vasc Surg 2004; 40: 634-9. 15472588 10.1016/j.jvs.2004.07.003

[5] Kusagawa H, Ozu Y, Inoue K, et al. Surgical management of recurrent varices secondary to reflux from subfascial veins or saphenous vein trunks in the thigh. Jpn J Phlebol 2016; 27: 323-30. (in Japanese)

[6] Perrin MR, Guex JJ, Ruckley CV, et al. Recurrent varices after surgery (REVAS), a consensus document. Cardiovasc Surg 2000; 8: 233-45. 10950599 10.1177/096721090000800402

[7] Kokubo M, Nozaka T, Takahashi Y. New method of flush saphenofemoral ligation that is expected to inhibit varicose vein recurrence in the groin: flush ligation using the avulsion technique method. Jpn J Phlebol 2017; 28: 11-6. (in Japanese) 10.3400/avd.oa.18-00086 PMC6200628 30402177

[8] Fischer R, Linde N, Duff C, et al. Late recurrent saphenofemoral junction reflux after ligation and stripping of the greater saphenous vein. J Vasc Surg 2001; 34: 236-40. 11496274 10.1067/mva.2001.115802

[9] Hartmann K, Klode J, Pfister R, et al. Recurrent varicose veins: sonography-based re-examination of 210 patients 14 years after ligation and saphenous vein stripping. Vasa 2006; 35: 21-6. 16535965 10.1024/0301-1526.35.1.21

[10] Kostas T, Ioannou C, Touloupakis E, et al. Recurrent varicose veins after surgery: a new appraisal of a common and complex problem in vascular surgery. Eur J Vasc Endovasc Surg 2004; 27: 275-82. 14760596 10.1016/j.ejvs.2003.12.006

[11] van Rij AM, Jiang P, Solomon C, et al. Recurrence after varicose vein surgery: a prospective long-term clinical study with duplex ultrasound scanning and air plethysmography. J Vasc Surg 2003; 38: 935-43. 14603197 10.1016/s0741-5214(03)00601-3

[12] Allegra C, Antignani PL, Carlizza A. Recurrent varicose veins following surgical treatment: our experience with five years follow-up. Eur J Vasc Endovasc Surg 2007; 33: 751-6. 17276095 10.1016/j.ejvs.2006.12.020

[13] Glass GM. Neovascularizaion in recurrence of the varicose great saphenous vein following transection. Phlebology 1987; 2: 81-91.

[14] Brake M, Lim CS, Shepherd AC, et al. Pathogenesis and etiology of recurrent varicose veins. J Vasc Surg 2013; 57: 860-8. 23343668 10.1016/j.jvs.2012.10.102

[15] Nyamekye I, Shephard NA, Davies B, et al. Clinicopathological evidence that neovascularization is a cause of recurrent varicose veins. Eur J Vasc Endovasc Surg 1998; 15: 412-5. 9633496 10.1016/s1078-5884(98)80202-5

[16] Recek C. Significance of reflux abolition at the saphenofemoral junction in connection with stripping and ablative methods. Int J Angiol 2015; 24: 249-61. 26648666 10.1055/s-0035-1546439 PMC4656171

[17] Nelzén O. Great uncertainty regarding treatment of varicose vein recurrence. Phlebologie 2014; 43: 13-8.

[18] Rass K, Frings N, Glowacki P, et al. Same site recurrence is more frequent after endovenous laser ablation compared with high ligation and stripping of the great saphenous vein: 5-year results of a randomized clinical trial (RELACS study). Eur J Vasc Endovasc Surg 2015; 50: 648-56. 26319476 10.1016/j.ejvs.2015.07.020

[19] Nelzén O. Reconsidering the endovenous revolution. Br J Surg 2016; 103: 939-40. 27168120 10.1002/bjs.10192

[20] El Wajeh Y, Giannoukas AD, Gulliford CJ, et al. Saphenofemoral venous channels associated with recurrent varicose veins are not neovascular. Eur J Vasc Endovasc Surg 2004; 28: 590-4. 15531192 10.1016/j.ejvs.2004.09.011

[21] Egan B, Donnelly M, Bresnihan M, et al. Neovascularization: an “innocent bystander” in recurrent varicose veins. J Vasc Surg 2006; 44: 1279-84; discussion, 1284. 17145430 10.1016/j.jvs.2006.08.017

[22] Stonebridge PA, Chalmers N, Beggs I, et al. Recurrent varicose veins: a varicographic analysis leading to a new practical classification. Br J Surg 1995; 82: 60-2. 7881959 10.1002/bjs.1800820121

[23] Bergan JJ, Ballard JI. Correction of superficial reflux. In: Gloviczki P, Bergan JJ, eds. Atlas of Endoscopic Perforator Vein Surgery. London: Springer-Verlag, 1998: 98-103.

[24] Rasmussen L, Lawaetz M, Serup J, et al. Randomized clinical trial comparing endovenous laser ablation, radiofrequency ablation, foam sclerotherapy, and surgical stripping for great saphenous varicose veins with 3-year follow-up. J Vasc Surg Venous Lymphat Disord 2013; 1: 349-56. 26992754 10.1016/j.jvsv.2013.04.008

[25] Hamann SAS, Giang J, De Maeseneer MGR, et al. Editorʼs choice five year results of great saphenous vein treatment: a meta-analysis. Eur J Vasc Endovasc Surg 2017; 54: 760-70. 29033337 10.1016/j.ejvs.2017.08.034

[26] Disselhoff BC, der Kinderen DJ, Kelder JC, et al. Five-year results of a randomised clinical trial of endovenous laser ablation of the great saphenous vein with and without ligation of the saphenofemoral junction. Eur J Vasc Endovasc Surg 2011; 41: 685-90. 21333560 10.1016/j.ejvs.2010.12.014

[27] Proebstle TM, Mohler T. A longitudinal single-center cohort study on the prevalence and risk of accessory saphenous vein reflux after radiofrequency segmental thermal ablation of great saphenous veins. J Vasc Surg Venous Lymphat Disord 2015; 3: 265-9. 26992304 10.1016/j.jvsv.2014.10.001

